# The sudden transition to synchronized online learning during the COVID-19 pandemic in Saudi Arabia: a qualitative study exploring medical students’ perspectives

**DOI:** 10.1186/s12909-020-02208-z

**Published:** 2020-08-28

**Authors:** Rehana Khalil, Ali E. Mansour, Walaa A. Fadda, Khaled Almisnid, Mohammed Aldamegh, Abdullah Al-Nafeesah, Azzam Alkhalifah, Osama Al-Wutayd

**Affiliations:** 1 Department of Family and Community Medicine, Unaizah College of Medicine and Medical Sciences, Qassim University, Unaizah, Saudi Arabia; 2 Department of Basic Medical Sciences, Unaizah College of Medicine and Medical Sciences, Qassim University, Unaizah, Saudi Arabia; 3 Department of Radiology, Unaizah College of Medicine and Medical Sciences, Qassim University, Unaizah, Saudi Arabia; 4 Department of Pediatrics, Unaizah College of Medicine and Medical Sciences, Qassim University, Unaizah, Saudi Arabia; 5 Department of Medicine, Unaizah College of Medicine and Medical Sciences, Qassim University, Unaizah, Saudi Arabia

**Keywords:** Sudden transition, Online learning, COVID-19 pandemic, Saudi Arabia, Qualitative study, Medical students, Perspective

## Abstract

**Background:**

The closure of educational activities in the Kingdom of Saudi Arabia due to the ongoing COVID-19 pandemic resulted in an unplanned shift from traditional learning to a setup that exclusively involves digital teaching and learning. Within this context, the present study aimed to explore undergraduate medical students’ perceptions regarding the effectiveness of synchronized online learning at Unaizah College of Medicine and Medical Sciences, Qassim University, Saudi Arabia.

**Methods:**

A qualitative study was conducted using virtual focus group discussions synchronously with the help of a discussion guide consisting of seven open-ended questions. Overall, 60 medical students were recruited using a maximum variation sampling technique; these students then participated in eight focus group discussions. All interviews were recorded, transcribed verbatim, and analyzed for thematic contents using the standard (Mayring, Kiger. M. E. and Braun.V) content analysis framework.

**Results:**

A thematic content analysis yielded four core themes: (1) educational impact, (2) time management, (3) challenges encountered, and (4) preferences for the future. The online modality was well-received, and all participants agreed that online sessions were time saving and that their performance was improved due to enhanced utility of time; however, they indicated that they encountered some challenges, including methodological, content perception, technical, and behavioral challenges during sessions and online exams. Most of the preclinical students preferred online learning for the upcoming academic years.

**Conclusion:**

Synchronized online classes were well-accepted by the medical students. This represents significant and promising potential for the future of medical education. The principles of the online learning model and learning outcomes should be rigorously and regularly evaluated to monitor its effectiveness.

## Background

The Covid-19 pandemic started in December 2019 in Wuhan, China and spread around the world rapidly within months. The pandemic affected all areas of life, including education. As the situation worsened, the global lockdown culminated in a lockdown of educational institutions. This closing of schools, colleges, and universities resulted in a stressful event for educational administration with highly limited options. The Saudi Ministry of Education announced online classes to continue the learning process in a safe and secure manner. All universities including medical universities were shifted to online learning within days [1]. This massive unplanned transition from traditional learning to an exclusively online learning setup has changed the methods of medical institutions in delivering the courses for their students. Medical graduates of the twenty-first century are exposed to online textbooks and modules with video lectures and computer-based exams. With this evolution in teaching modalities, a “flipped classroom” model for learning has been adopted by many medical schools around the world [2, 3]. Although these experiences of medical institutions are not similar, this can at least help them in accepting synchronized online models in this critical period.

Online learning is classified as synchronous or asynchronous. Synchronous technology allows for “live” interaction between the instructor and the students (e.g., audioconferencing, videoconferencing, web chats etc.) while asynchronous technology involves significant delays in time between instruction and its receipt (e.g., E-mail, earlier video recording, discussion forums etc.) [4].

It has long been acknowledged that online instructional methods are an efficient tool for learning [5]; however, online learning can be challenging for students because of the limited non-verbal communication. Other aspects, such as students’ and professors’ interactions, accessibility of materials, and time management, can also affect the opinions of online education participants [6]. To assess students’ performance in an online course, a representative set of face-to-face courses should be compared to a similar set of online courses. This strategy was adopted in a study that involved utilizing a dataset of hundreds of courses being taught at 23 colleges of Virginia’s community college system; the authors found that students’ performance was worse in online courses with respect to both course persistence and end-of-course grades [7].

Online learning in medical education can lead to more effective and easier access to a greater quantity of information, especially in uncertain global situations such as pandemics [8]. There is no doubt that the current COVID-19 pandemic has augmented the focus on online learning in education, but we anticipate that in the future, this shift will prove to be a permanent trend in medical education. The methods used by Unaizah College of Medicine and Medical Sciences included lectures, case discussions, 4-box case analysis, clinical case discussions, online seminars, and dry labs (online laboratory demonstrations). The largest proportion was covered through lectures, which was about 60%, while case discussions (including 4-box case analysis and clinical case discussions) consisted of 20%, Online seminars consisted of 10% and dry laboratories made about 10% of courses. We conducted this qualitative study to assess the effectiveness of these techniques and [9, 10] the barriers to medical students’ engagement in online learning [9]. The foremost goal of our exploratory study is to determine medical students’ perceptions and satisfaction level regarding synchronous online learning methods implemented in courses during their second semester as an emergency intervention during the ongoing Covid-19 pandemic in the Kingdom of Saudi Arabia.

## Methods

### Study design

We employed an online focus group qualitative design for our study for numerous reasons. Firstly, this design enables an interactive and in-depth exploration of respondents’ experiences [11, 12]. Secondly, the group process can help individuals to clarify views that might not emerge from a one-on-one interview. In addition, it can uncover extensive opinions that individuals hold about an issue, as well as perceptive differences among individuals and groups [12]. Therefore, to effectively address the objectives of our study, focus group discussions were the most suitable choice. The discussions were conducted online because of the ongoing Covid-19 pandemic and social distancing in the Kingdom of Saudi Arabia and because this approach was cost-effective [13]. It also ensured a detailed probing of the perceptions, expectations, and difficulties and was timed so as to generate recommendations for improvements and hypotheses that can be tested in future research.

### Study participants

Participants were purposely sampled. To ensure sufficient diversity of opinion among groups, medical students in their first year through final year (attending online courses during their second semester) studying at Unaizah College of Medicine and Medical Sciences, Saudi Arabia, were recruited using a maximum variation sampling technique [14]. Roll-call lists of medical students were used as a sampling frame to select the participants. The class leaders of the first-year class through the final-year class were contacted. The reason for selecting class leaders first was that they had direct contact with the instructors and their classmates. As a result, they acted as connectors, and other participants were selected with the help of those class leaders. Upon contact, each student had been introduced to the background of our study and was invited to participate. Overall, 60 students participated in eight focus group discussions, and each group had seven to eight participants. A satisfactory (40/60) male-to-female participant ratio was maintained in discussions. Focus group discussions were continued unless the saturation of new information was encountered.

### Procedure

The students who opted to participate in our study received an e-mail with information about the use of the online focus groups’ forum along with a discussion guide to generate discussion. Each of them was given a personal log-in name and password to access the online forum. For data collection, only the moderators had access to the forum. The anonymity of statements in the transcripts and in the final report was ensured, as well as the confidentiality and security of the data. Because data collection was done through the Internet, participants provided informed consent by clicking a button after having read all relevant information.

### Study setting and data collection

The electronic focus group synchronous discussions were based on a discussion guide which was developed by two authors (OW and AM), in consultation with experts for this study. The guide was revised and approved by all authors, and piloted with four students. It consisted of seven open-ended questions, allowing participants to discuss as many elements as possible. The focus group discussions were conducted during the months of April and May 2020 by dual moderators.

The forum for each group started with an introduction and proceeded with questions for participants about their experiences with online learning, the differences between online learning and on-campus-learning, difficulties encountered, challenges, level of satisfaction with online evaluation, and preferences regarding learning modalities for their next academic year. All students in each focus group simultaneously took part in a live synchronous session for discussion by logging into the website. The students used an online conferencing website, and their discussions were audio-recorded. Two moderators were present; one ensured the smooth progression of the focus group sessions, and the other made sure that the topics in questions were all covered. Dual-moderator focus groups resulted in highly productive sessions. Each group discussion took between 90 and 120 min. During the final two focus groups discussions, moderators agreed that saturation had been reached and that the inclusion of more respondents would thus be unnecessary.

### Data analysis

Discussions were recorded, transcribed verbatim, and analyzed by summarizing content analysis developed by Mayring [15], Kiger. M. E [16], and Braun. V [17]. This helped to condense the data into essential content in a systematic manner guided by sequential steps. The main themes of the data were based on the discussion questions. An inductive process was used for analysis and to assign a code for each meaningful sentence and then gather similar codes in overarching sub-themes. Finally, similar sub-themes were grouped together under a main theme reflecting its sub-themes. The data was analyzed and coded by all authors. Two authors performed a preliminary analysis, with the remaining authors acting as second coders for the data. Subsequently, the initial coding was reviewed and compared. It was then contemplated and refined until a consensus was achieved among all authors, which led to a more representative coding scheme, sub-themes, and themes.

## Results

In total, eight online synchronous focus group discussions were conducted. Each group included seven to eight participants. There were a total of 60 respondents, and more than half (60%; *n* = 36) of the participants were female. The approximate male/female students’ distribution within Unaizah College of Medicine and Medical Sciences is 44/56. Half of the sample participants (50%; *n* = 30) were between 21 and 22 years old. Almost three fourth of the participants (72%, *n* = 43) resided within the city (Unaizah) where their medical college campus was located, as indicated in Table 1.

**Table 1:** Characteristics of the study respondents (*N* = 60)

Characteristics	Frequency (n)	Percentage (%)
Age (years)		
< 21	7	12
21–22	30	50
23–24	17	28
> 24	6	10
Gender		
Male	24	40
Female	36	60
Educational Status		
Pre-Clinical Years		
Premed-2 (Year-1)	7	12
MD-1 (Year-2)	15	25
MD-2 (Year-3)	15	25
Clinical Years		
MD-3 (Year-4)	8	13
MD-4 (Year-5)	15	25
Residence		
Within City	43	72
Outside City	17	28

During the analysis, sub-themes were identified and classified under four major themes, which are summarized below with relevant quotes from the participants.

Four core themes included the following: (1) educational impact (2), time management (3), challenges encountered (4), and preferences for future as indicated in Table 2.

**Table 2:** Summary of themes and sub-themes

	Themes	Sub-themes	Description	Example
1	Educational impact	Content understanding	Educational improvement due to better understanding of information	“Yes, there is no doubt that online classes are better than campus-based classes because recorded lectures are very helpful. It helped me a lot in my academic progress this year.”
		Content perception challenges	Difficulties in understanding of the online delivered information due to variations in demand of content reception by learners	“I faced difficulty in understanding some of the lectures, especially those containing x-rays, were not clear in the online sessions.”
2	Time management	_	Improved time organization and utility due to online learning	“Online sessions provided me with a great time to study and I experienced better time management.”
3	Challenges encountered	Methodological challenges	Quality assurance issues in the content delivery and implementation issues of the online learning	“There were a lot of lectures scheduled in one day! Honestly speaking, I didn’t get time to study them well...”
		Technical challenges	Difficulties experienced due to technological hindrances of internet connectivity and poor utility of online tools	“Slow internet connectivity and communication software failure were among frequent technical issues ....”
		Behavioral challenges	Barriers in adoption of online learning influenced by the individual personality characteristics	“It’s not suitable for me because I am a visual and kinesthetic learner. I must admit that even though online classes helped me in raising my marks, ...”
4	Preferences for future	_	Students’ choices of learning modalities for their next academic year	“I would like to continue online classes if system is fool-proof and well prepared before we start using it again. I mean the technical part.”

### Theme 1: educational impact

The educational impact of online courses identified by the respondents of our study are discussed under two sub-themes, including (1) content understanding and (2) content perception challenges.

The first sub-theme concerned the experience of content understanding. Although our study participants held different opinions about content understanding through online classes, two-thirds of them agreed that it works better for some disciplines, which they elaborated upon in the following statements:

“In my opinion, online classes proved to be an excellent opportunity for theoretical subjects like basic sciences but not suitable for clinical subjects like clinical skills etc.”

Experiences of clinical students were different from preclinical students. Some of the fourth-year and fifth-year students found online classes highly beneficial, and they expressed their views as follows:

“Some online learning courses are very useful, such as radiology and we were really benefited from online sessions like seminars and case discussions.”

“Online classes provided me with great opportunities to focus on applied courses like radiology, forensic medicine.”

Almost all of the students regarded it as an opportunity to utilize recorded lectures to better understand and master the content. They revealed the following:

“Lectures recording option in online learning, benefited us a lot! I can listen to the lecture again and again, at my convenience and can make notes very easily. I consider it, a wonderful experience at the end of this academic year.”

“Yes, there is no doubt that online classes are better than campus-based classes because recorded lectures are very helpful. It helped me a lot in my academic progress this year.”

The second sub-theme which emerged concerned content perception challenges, which encompass variations in demand of content reception and learning by different types of learners. Under this sub-theme, participants’ expressed the following views:

“I faced difficulty in understanding some of the lectures, especially those containing x-rays, were not clear in the online sessions.”

“In my opinion, non-verbal communication like eye contact with the instructor is essential to establish learning process. Campus learning allows discussion among students which is very helpful for clearing a lot of concepts.”

“For me, online lectures are useful only for theoretical explanation of the content but I miss clinics! Absence of the clinical practice and labs is problematic for me to master the practical concepts.”

### Theme 2: time management

Participants generally referred to the theme of time management as a dominant perspective, and they all agreed that online sessions saved time for them and that their performance had improved as a result. This is made evident in the following statements:

“Online sessions provided me with a great time to study and I experienced better time management.”

“I live away from the college campus and it usually takes two hours daily to reach college. So, these online classes saved my time by reducing my daily exertion of going to campus and coming back home.”

They experienced more contentment with online courses because of their physical ease and because being within their comfort zone reduced their anxiety. They expressed their views in the following statements:

“Online classes had a positive effect on me in terms of saving time and effort, by reducing the campus-based distractions like compulsive participation in conversions, adjustment with unavoidable noise and waste of time in finding a proper place to study alone.”

“I thoroughly enjoyed online courses. I used to choose a comfortable place at my home for my online classes and it also gave me a chance to relax at my convenience. It really saved my time and effort to study well.”

“Online learning has a positive impact on my sleep pattern and I felt more comfortable and balanced.”

Most of the participants were satisfied with online courses because they were able to spend more time with their families. One stated the following:

“I experienced an entirely new method of time management with online sessions. Online classes allowed me to save time for my studies and I found more time to sit with my family and enjoy quality refreshing time, whenever I wanted to take a break between my studies.”

### Theme 3: challenges encountered

The participants encountered some challenges and identified barriers to the acquisition of knowledge through online courses. These are discussed under the following three sub-themes here: (1) methodological challenges (2), technical challenges (4), and behavioral challenges.

The first sub-theme, which can be regarded as a negative aspect from the perspective of participants, was methodological challenges, which included quality assurance issues in the content delivery of the lectures and implementation issues. Adjustment and engagement in the new system was perceived as a barrier to the development and implementation of online learning, and there were multiple problems related to the duration and arrangement of learning sessions. Some participants expressed their ideas about these issues as follows:

“It felt like, some of the online lectures were unnecessarily allotted long time! The sequence of the online lectures was also suboptimal and there was a frequent change of lecture timing. I felt difficulty in connecting the information out of content heaps.”

“There were a lot of lectures scheduled in one day! Honestly speaking, I didn’t get time to study them well. Most of the lectures’ content was huge and was covered within limited time and some of the instructors were not committed to the time of the lecture. It felt like we are taking lectures whole day.”

“The duration of some lectures was very long! Instructors were not committed to the allotted time of the lectures. Some instructors were late for the lectures and did not start it on time. The lectures were frequent during the day and at night.”

“I noticed a serious communication gap between students and instructor. At time I needed more clarification for some lecture content but those points were left unexplained by the instructors because of miscommunication. I missed direct discussions with my instructors and my classmates during campus-classes.”

Technical challenges faced by the participants are discussed under the second sub-theme. This includes all essential technical elements, such as internet connectivity and the use of online tools. Participants listed many technical issues which they faced during online sessions, as mentioned in the following statements:

“Slow internet connectivity and communication software failure were among frequent technical issues which I faced during whole course. Second big issue was, most of our instructors have no experience in delivering online lecture. There was a wastage of time every day because of technical problems.”

“I used to face very frequent internet disconnection during online lectures daily and it was very hard for me to follow lectures with instructors.”

“My participation was greatly affected by issues like delayed download the lectures and internet lagging! The technical problems like troubled sound due to pressure on the internet software was very common during online sessions.”

“Some instructors were not in a habit of checking their microphones before starting their lectures, so there was interrupted voice which led to unnecessary inconvenience and botheration.”

“Sometimes instructor’s voice was not clear and they didn’t use appropriate explanatory tools given in the online software. It led to wastage of time which could be otherwise avoided.”

Behavioral and acceptability challenges are discussed under our third sub-theme, which encompasses negative attitudes towards the adoption of new modes of learning. Participants’ perspectives about their acceptability of online learning is quite discernible from the following statements:

“It’s not suitable for me because I am a visual and kinesthetic learner. I must admit that even though online classes helped me in raising my marks, but it did not help me in upgradation of my knowledge.”

“Even though, I managed to cope up with online teaching but I desperately missed body language like eye to eye contact with instructors and writing on the board. I missed active interactive sessions like team-based learning sessions, peer instruction skills and discussion among students. I prefer to study within campus ambit.”

“Most of the times, I did not find a suitable place at home for taking my online classes and I felt like environment is not suitable at home for attending online lectures.”

“My family did not realize that I am seriously busy in learning through online system and that put a lot of pressure on me.”

“If online lectures are continued then, I am afraid that it will lead to laziness and its negatively affecting my performance, as it takes me longer to study the content.”

“The best thing about online lectures was “recorded lectures”, but at the same time the drawback was, I could not link the theoretical concepts together through recorded lectures. It simply doesn’t work for me.”

“Online learning kept me away from my family, and it feels like I am always busy in my lectures and studies.”

“While learning through online sessions, I had no time to enjoy my social life. I feel like I am busy all the time with my lectures and I lost interaction with my classmates.”

### Theme 4: preferences for the future

The students were asked about their preferences regarding their next academic year. Mixed responses were conveyed. The majority of them preferred online learning, while others, especially clinical students, expressed interest in the continuation of campus-bound classes and live clinical participation. The statements in favor of campus-based classes included the following:

“It is time saving to study together to master some difficult concepts. Some lectures that need active discussion and eye to eye contact with instructors while others need joint study like team-based learning skills.”

“Courses that require practical application, such as women and children health, cannot be taught though online sessions.”

“It was an unpleasant experience. Please do not repeat it in any course of next academic year. In my opinion, college campus and clinics are most suitable for learning because it provides us with a special learning environment.”

“Well, it did not help me much in my academic progress. Clinical practice and direct learning at campus though live lectures is better, as eyes to eye contact with the instructors helps in better understanding of content.”

Online experience of learning was preferred by some students, but they suggested some conditions in case of its continuation. The following statements elaborated upon this:

“I prefer online learning, provided the doctor should use the online teaching tools like writing on screen, highlighting the text etc.”

“I prefer to continue with online learning in upcoming academic year, only in the fields of scientific research and forensic medicine.”

“I agree to continue online sessions for some courses like radiology but it cannot be applied in all courses, especially clinical courses. Excellent in theoretical courses but unsatisfactory experience in practical courses.”

“Online learning is better than studying in college campus and is preferable for female students like me who lives outside Unaizah.”

“I would like to continue online classes if system is fool-proof and well prepared before we start using it again. I mean the technical part.”

“Online learning is excellent and saves time for studying. I suggest that there should be one day per week specified for online learning to cover some theoretical courses but not for whole year’s courses.”

## Discussion

Our research study examined undergraduate medical students’ perspectives and preferences regarding online modules of synchronized learning. The concept of online classes is not new in the Kingdom of Saudi Arabia, in relation to online synchronized learning in medical education at the undergraduate level, no research was previously conducted in the Kingdom.

In our analysis, we have identified the factors influencing the medical students’ assent or dissent in relation to synchronized online learning. Assenting students in our study were motivated by the benefit of mastering the content in less time compared to campus learning. They utilized updated educational technologies which fulfilled the requirements of adult learners by promoting active and student-centered learning. This instructional method helped empower learners in controlling their educational needs constructively and provides them with structured guidance for self-directed learning [18]. Eventually, this enables students to utilize their time productively to achieve their individual goals. Additionally, most of the students in this study found more time to spend with their families and to improve their sleep pattern. However, some students mentioned some negative factors related to managing their time. The overall satisfaction level with online learning was found to be high in our study, and students’ attitudes were quite positive towards online learning as a new teaching modality relative to traditional didactics. This finding is consistent with previous research on online learning modalities [19–38].

However, our study assessed the individual learning experiences using online modules, and we found that the use of online learning is more productive within the context of particular medical disciplines, such as for basic medical sciences or preclinical subjects; this made it difficult to compare its relative effectiveness with clinical disciplines [19, 20]. That is why more preclinical students preferred online learning for their future academic years, as compared to clinical students who participated in our study. Conversely, Cardall S. conducted a study in 2008 which concluded that preclinical students preferred to have live lectures when given an option; the authors agreed that online lectures are an efficient method for the acquisition of knowledge [39].

According to our study participants, the missing element in the effectiveness of online learning was clinical practice. Nothing can replace seeing a patient because clinical experience and human interaction are extremely important for the practice of medicine. However, online learning may serve as an efficient resource for clinical students if the method is upgraded through the integration of modalities such as virtual simulation technologies and computer-based models of real-life processes. This can produce multiple benefits for clinical learners by providing controlled opportunities to practice rare and critical events in safe environments excluding the risk to patients. An instructional technology, such as a computer-based virtual patient program designed to simulate real-life clinical scenarios, can be useful for clinical learners to facilitate history-taking and physical examination and can encourage diagnostic and therapeutic decision-making [18].

Although the themes that emerged from this study support the notion that “online learning works for medical students,” this does not imply that online learning can completely replace in-person live sessions. Our study participants encountered numerous challenges in adopting online learning. One of the most common barriers was technical insufficiency, including poor internet connectivity and deficits in educators’ basic computer skills. The themes identified in our study complement numerous previous studies [40–42]. The high-yielding technological use in medical education depends on the faculty’s readiness and expertise to employ the technology to facilitate learning. Training physicians with these skills need a divergence from traditional teaching methodology. Hence, it appears that training in educational technology mastery is a neglected competency in faculties which should be mandatory for the enhancement of medical universities [2].

Aside from technical issues, our study participants’ experiences were influenced by the individual characteristics of students themselves, such as their learning styles, acceptance of new learning modalities, and levels of engagement in online classes [43–45]. Other barriers included quality assurance issues in the implementation of online learning by the institution. This finding is consistent with a study performed by Bediang et al. in 2013, in which the authors concluded that one of the most imperative means for implementing online modules is encouraging collaboration among all departments and stakeholders. An organized and clear institutional approach is required to formulate a well-regulated and efficient system which can facilitate the adoption of structured methodologies by faculty members while implementing an online learning module [10].

A lack of non-verbal communication by instructors was also identified as a significant challenge for our study participants. According to communication theorists, verbal messages are conveyed through words, whereas nonverbal messages are conveyed beyond the actual meaning of words, which typically complements the spoken words [46]. The psychological closeness which a student may feel with their instructor is primarily based upon the instructor’s nonverbal clues. Nonverbal communication, such as eye contact, gestures, and posture, comprise a major part of all communications.

## Conclusion

Our study concludes that synchronized online learning was well-received by the medical students. At the same time, some challenges for our study participants included technical issues, individual behavioral characteristics, institutional methodology barriers, and the absence of non-verbal clues. Moreover, preclinical students were more likely to opt for online lectures as their preference for the next academic year compared to clinical students.

### Limitations

There are some limitations to this study. The findings of this study cannot be generalized because it was conducted in only one medical school. Although four core themes and a number of subthemes emerged from our study, the authors are aware that there must be other potential strengths and weaknesses of this modality for undergraduate medical students. Secondly, to ensure the effectiveness of online learning modules for undergraduate medical students, the principles of online learning model and learning outcomes should be rigorously and regularly evaluated.

### Recommendations

The findings of our research support the evidence regarding the effectiveness of online learning for medical students; however, it is important to realize that it is not the only mode of transferring efficient knowledge. Of course, there are other teaching modalities and clinical teaching which are essential to implement as compulsory parts of an ideal undergraduate medical education model. Thus, the online module’s predominately synchronized learning represents a meaningful and promising potential for the future of medical education and can be integrated into the curriculum to enhance the effectiveness for lifelong learning.

There is a need for research to identify other effective online and offline teaching modalities and to formulate a meticulous model through the integration of an optimal proportion of online learning in undergraduate medical education.

## Supplementary information

**Additional file 1.**

## Data Availability

Data sharing is not applicable to this article.

## Abbreviations

COVID-19: Coronavirus disease 2019
MD: Doctor of Medicine
