# Supplementary material for: The sudden transition to synchronized online learning during the COVID-19 pandemic in Saudi Arabia: a qualitative study exploring medical students’ perspectives
Source: BMC Med Educ. 2020 Aug 28;20:285. doi: 10.1186/s12909-020-02208-z (PMC7453686; doi:10.1186/s12909-020-02208-z)
Supplement: Supplementary file 1 — Additional file 1. [file 12909_2020_2208_MOESM1_ESM.docx]

**Additional file 1:**

**The sudden transition to synchronized online learning during the COVID-19 pandemic in Saudi Arabia: A qualitative study exploring medical students’ perspectives**

**Focus Group Discussion Guide**

1. Tell me about your experience with synchronized online learning. Was it good or bad? Please tell me what you like or don’t like about it?
2. What are the differences between synchronized online learning and direct learning in the college campus?
3. What is your opinion about continuation of synchronized online learning in your next academic year?
4. Are you satisfied with the synchronized online learning and evaluation? Elaborate your answer with reasons.
5. What were your concerns during synchronized online learning?
6. How far did synchronized online learning affect your family and daily life?
7. Is there anything further you would like to discuss that we did not ask you regarding the synchronized online learning. How helpful was it for your academic progress?
